# Bariatric surgery and calcifediol treatment, Gordian knot of severe-obesity-related comorbidities treatment

**DOI:** 10.3389/fendo.2023.1243906

**Published:** 2023-10-06

**Authors:** Aura D. Herrera-Martínez, Laura L. S. Castillo-Peinado, María J. Molina-Puerta, Alfonso Calañas-Continente, Antonio Membrives, Juan Castilla, Marta Camacho Cardenosa, Antonio Casado-Díaz, María A. Gálvez-Moreno, Manuel D. Gahete, José Manuel Quesada Gómez, Roger Bouillon, Feliciano Priego-Capote, Raúl M. Luque

**Affiliations:** ^1^ Maimonides Institute for Biomedical Research of Cordoba (IMIBIC), Córdoba, Spain; ^2^ Endocrinology and Nutrition Service, Reina Sofia University Hospital, Córdoba, Spain; ^3^ Department of Analytical Chemistry, University of Córdoba, Córdoba, Spain; ^4^ Chemical Institute for Energy and Environment (IQUEMA), University of Córdoba, Córdoba, Spain; ^5^ CIBER Fragilidad y Envejecimiento Saludable (CIBERFES), Instituto de Salud Carlos III, Madrid, Spain; ^6^ General Surgery Service, Reina Sofia University Hospital, Córdoba, Spain; ^7^ CIBER Fisiopatología de la Obesidad y Nutrición (CIBERobn), Córdoba, Spain; ^8^ Department of Cell Biology, Physiology, and Immunology, University of Córdoba, Córdoba, Spain; ^9^ Clinical and Experimental Endocrinology, Department of Chronic Diseases and Metabolism, Catholic University of Leuven, Leuven, Belgium

**Keywords:** obesity, bariatric surgery, inflammasome, vitamin D, comorbidities

## Abstract

**Background:**

Obesity (OB) is a chronic metabolic disease with important associated comorbidities and mortality. Vitamin D supplementation is frequently administered after bariatric surgery (BS), so as to reduce OB-related complications, maybe including chronic inflammation.

**Aim:**

This study aimed to explore relations between vitamin D metabolites and components of the inflammasome machinery in OB before and after BS and their relations with the improvement of metabolic comorbidities.

**Patients and methods:**

Epidemiological/clinical/anthropometric/biochemical evaluation was performed in patients with OB at baseline and 6 months after BS. Evaluation of i) vitamin-D metabolites in plasma and ii) components of the inflammasome machinery and inflammatory-associated factors [NOD-like-receptors (NLRs), inflammasome-activation-components, cytokines and inflammation/apoptosis-related components, and cell-cycle and DNA-damage regulators] in peripheral blood mononuclear cells (PBMCs) was performed at baseline and 6 months after BS. Clinical and molecular correlations/associations were analyzed.

**Results:**

Significant correlations between vitamin D metabolites and inflammasome-machinery components were observed at baseline, and these correlations were significantly reduced 6 months after BS in parallel to a decrease in inflammation markers, fat mass, and body weight. Treatment with calcifediol remarkably increased 25OHD levels, despite 24,25(OH)_2_D_3_ remained stable after BS. Several inflammasome-machinery components were associated with improvement in metabolic comorbidities, especially hypertension and dyslipidemia.

**Conclusion:**

The beneficial effects of vitamin D on OB-related comorbidities after BS patients are associated with significant changes in the molecular expression of key inflammasome-machinery components. The expression profile of these inflammasome components can be dynamically modulated in PBMCs after BS and vitamin D supplementation, suggesting that this profile could likely serve as a sensor and early predictor of the reversal of OB-related complications after BS.

## Introduction

Obesity (OB) is characterized by an excess amount of body fat; it has an important negative impact on health and quality of life (1). OB is a major public health problem due to its growing incidence, high frequency of comorbidities, and mortality (2, 3). Metabolic comorbidities are observed in 10%–30% of patients (4), especially type-2 diabetes (T2DM), hypertension (HT), heart disease, high total cholesterol and triglycerides, stroke, and non-alcoholic fatty liver disease (NAFLD) (5–7).

Bariatric surgery (BS) is a well-recognized treatment for severe OB and generates superior outcomes compared with non-surgical methods (8). BS induces substantial and sustained weight loss through a variety of mechanisms (9–12), which are accompanied by reduction in obesity-related comorbidities and improvement in quality of life (13, 14). In clinical practice, the measurement of the total blood concentration of 25OHD is the marker of the nutritional status of the vitamin D endocrine system (VDES) (15).. Obesity is consistently characterized by lower 25(OH)D blood levels due to several mechanisms including volumetric dilution, adipose tissue (AT) sequestration, impaired hepatic 25-hydroxylation, altered VDES metabolism in AT, and some unknown additional mechanisms (16–20).

Several external factors influence serum/plasma 25(OH)D levels such as season, geographical location, ethnicity, and sunlight exposure (21). Additionally, endogenous factors such as OB, starvation, diabetes, and glucocorticoids have a major influence on cytochrome P450 Family 2 subfamily R member 1 (CYP2R1) activity, affecting the production, and in consequence the circulating levels of 25(OH)-D (22). In this context, total serum 25(OH)-D may incorrectly reflect VDES status. It has been proposed that the ratio of serum 24,25-dihydroxyvitamin D (24,25(OH)2 D to 25(OH)D (vitamin D metabolite ratio [VMR]) could represent a new and more reliable biomarker for assessing vitamin D status in processes such as vitamin D deficiency, idiopathic childhood hypercalcemia, and chronic kidney disease, and even assessing the entire VDES metabolome, idiopathic childhood hypercalcemia, and chronic kidney disease (23), or even to assess the entire VDES metabolome by liquid chromatography coupled to tandem mass spectrometry (SPE–LC–MS/MS) as the technique of choice (24).

Deficiency of 25(OH)-D has been associated with metabolic comorbidities including NAFLD, T2DM, insulin resistance, and metabolic syndrome (25–27). In this context, several studies have reported the influence of vitamin D supplementation on reversion of metabolic comorbidities with contradictory results (28, 29). Recent studies have shown that low levels of 25(OH)-D have been associated with a higher prevalence of infections and autoimmune diseases, while adequate levels have been correlated to protection of these processes (30, 31).

OB is characterized by the presence of low-grade chronic inflammation, due to increased pro-inflammatory cytokine production by macrophages and adipocytes. Multiple studies have shown that in the presence of abdominal adiposity, vitamin D deficiency is linked to inflammation and decreased insulin sensitivity (32).

VDES is a key modulator of immune function and inflammation, the active metabolite of the system reduces adipocyte chemokine and cytokine release, and monocyte chemotaxis (33). Its effects on the systemic and tissue-specific inflammatory response have been attributed to a variety of factors, including suppression of the nuclear factor-κB (NF-κβ pathway), T-helper cell anti-inflammatory activation, reduction in toll-like receptor 4 (TLR-4) expression (which reduces the differentiation of dendritic cells), and modulation of inflammasome activation (34, 35).

Inflammasomes are multimeric protein complexes that detect pathogenic microorganisms and sterile stressors, activate highly pro-inflammatory cytokines, and are, in consequence, responsible for innate immune system response (36, 37). Inflammasome have germline-encoded pattern-recognition receptors (PRRs), which recognize the presence of unique microbial components [pathogen-associated molecular patterns (PAMPs)], including bacterial flagellin or damage-associated molecular patterns (DAMPs), such as uric acid crystals, which are generated by endogenous stress (38); in consequence, inflammasome activates the innate immune system (39) in response to infection and/or to repair damaged tissues (40).

Remarkably, the activity of inflammasome components is regulated by different regulatory proteins, metabolic pathways, and a regulatory mitochondrial hub (37, 41). In consequence, the activation of these inflammasome components leads to the secretion of diverse inflammatory cytokines and key receptors in immune cells, thus inducing the activation of inflammatory cascades, which can lead, in some cases, to cell-cycle alterations and DNA damage (36, 41).

Dysregulation of inflammasome has been associated with several inflammatory disorders including Alzheimer’s disease, autoinflammatory diseases, and OB-related comorbidities, including T2DM, NAFLD, and atherosclerosis (36, 37). A preliminary study of our group has reported dysregulation of several components of inflammasome in patients with OB and their relation with the presence of metabolic comorbidities (42).

Considering the link between vitamin D, inflammation, inflammasome, and OB, the aim of the present study was to investigate the clinical relation between VDES status, inflammasome activation, and reversal of metabolic comorbidities in patients with OB who underwent BS and received oral supplementation with calcifediol (25(OH)3. Peripheral blood mononuclear cells (PBMCs) were used, since gene expression patterns are commonly reflected in these cells and are closely related to the molecular profile of the disease (43). Specifically, the gene expression levels of four groups of components of the inflammasome machinery, namely, 1) NLRs or NOD-like receptors, 2) regulators of inflammasome activation, 3) cytokines and inflammation/apoptosis-related components, and 4) cell-cycle and DNA-damage regulators, were analyzed. Additionally, we measured VDES metabolites using SPE–LC–MS/MS, which has been reported as an accurate and robust method for measuring vitamin D metabolites in human serum (23, 44). The putative relations between gene expression levels of the inflammasome machinery with vitamin D metabolites and the clinical evolution of metabolic and inflammatory comorbidities, associated with OB, after BS were explored.

## Materials and methods

### Patients

This study was approved by the Ethics Committee of the Reina Sofia University Hospital (Cordoba, Spain; registration code CB01072018) and was conducted in accordance with the Declaration of Helsinki and according to national and international guidelines. This is a prospective open-label study, wherein a written informed consent was signed by every individual before inclusion into the study. A total of 40 consecutive patients who underwent BS were included. Clinical records were used to collect full medical history of all patients (demographic and clinical characteristics of patients are summarized in **Table 1**). All patients were managed following available clinical guidelines and recommendations (45–49). They received medical treatment with oral calcifediol in variable dose in order to reach blood levels >30 ng/dL (0.266 mg every 10, 15, 21, or 30 days). Clinical follow-up was performed by the same clinician in all cases at baseline (before surgery) and 6 months after BS. Body composition was evaluated using a multi-frequency bioimpedanciometer (Tanita MC-780MA, Barcelona, Spain), and waist circumference was measured at minimal expiration. Blood samples were obtained at baseline and 6 months after BS from all patients to obtain plasma, serum, and PBMCs. Regular physical exercise and health style education are regularly advised to all patients; specific evaluation of the patient adherence was not performed.

**Table 1:** Clinical characteristic of the evaluated patients (n=40).

Characteristic	Baseline	6 months after bariatric surgery	*p*°
Gender (%)			
Female	55 (22/40)		
Male	45 (18/40)		
Age (years)	45.23 ± 10.65		
BMI (kg/m2)	46.7 (41–52)	36.6 (31–39)	<0.001
Body weight	122.5 (82–186)	91 (64–137)	<0.001
Fat mass (%)	45 (43–47)	36 (22–46)	<0.001
Fat mass (kg)	54 (26–87)	35.5 (17–52)	<0.001
Lean mass (%)	54.2 (47–61)	60.8 (52–73)	0.03
Lean mass (kg)	67 (45–97)	59.5 (24–92)	0.004
Water (%)	38.9 (33.48)	45.5 (37–56)	<0.001
Water (kg)	52 (35–73)	44.4 (29–65)	<0.001
Abdominal perimeter	134 (114–154)	117 (91–146)	<0.001
Serum 25–OHD (ng/dl)*	17.4 (11–24)	32.1 (25–40)	<0.001
Serum RCP (g/dl)	6.8 (4–10)	2.1 (.03–4)	<0.001
Weight loss (%)	25.7 (20–28)		
Type of surgery			
Sleeve (%)	27.5 (11/40)		
Bypass (%)	72.5 (29/40)		

*Determined using chemiluminescence with acridine ster.

### Chemicals and reagents

Mass spectrometry grade ammonium formate and formic acid (FA) were acquired from Sigma (Sigma–Aldrich, St. Louis, MO, USA) as ionization and sorbent activation agents; methanol and acetonitrile (ACN) from Scharlab (Barcelona, Spain) and deionized water (18 mΩ cm) from a Millipore Milli-Q water purification system were employed for the preparation of chromatographic mobile phases and SPE solutions.

Analytical standards of vitamin D_3_, 25-hydroxyvitamin D_3_ [25(OH)D_3_], 1,25-dihydroxyvitamin D_3_ [1,25(OH)_2_D_3_], 24,25-dihydroxyvitamin D_3_ [24,25(OH)_2_D_3_] and their deuterated internal standards (ISs), vitamin D_3_-d_3_, 25(OH)D_3_-d_3_, 1,25(OH)_2_D_3_-d_3_, and 24,25(OH)_2_D_3_-d_6_, were purchased from Sigma-Aldrich. 1,24,25-Trihydroxyvitamin D_3_ [1,24,25(OH)_3_D_3_] analytical standard was obtained from Quimigen S.L. (Madrid, Spain), but no IS for this analyte was available. According to endogenous concentration ranges of these metabolites found in blood samples, a multistandard working solution was prepared: vitamin D_3_ at 2.5 μg mL^–1^, 25(OH)D_3_ at 15 μg mL^–1^, 24,25(OH)_2_D_3_ at 0.5 μg mL^–1^, 1,25(OH)_2_D_3_ at 15 ng mL^–1^, and 1,24,25(OH)_3_D_3_ at 50 ng mL^–1^. Since the presence of matrix effects has been proven in LC–MS/MS analyses of vitamin D_3_ and its metabolites in blood samples [2,16], a working solution of ISs was used, in order to correct results variations due to this phenomenon, at the following concentrations: vitamin D_3_-d_3_ and 25(OH)D_3_-d_3_ at 625 ng mL^–1^, 24,25(OH)_2_D_3_-d_6_ at 125 ng mL^–1^, and 1,25(OH)_2_D_3_-d_3_ at 7.5 ng mL^–1^.

### Online SPE–LC–MS/MS determination

Sample preparation step consisted of thawing plasma samples at room temperature, centrifuging (4°C) at 20,000×*g* for 10 min and sterilizing the resulting supernatant fraction by filtration. Then, aliquots of 240 μL were pretreated by adding 10 μL of ISs working solution to each one followed by SPE after shaking in vortex for 2 min. Regarding SPE stage, activation of cartridge’s sorbent was achieved by adding 6 mL of methanol and conditioning and equilibration of cartridges by adding 8 mL of the loading sample solvent mix as recommended by the manufacturer. However, different loading sample and interferents clean-up volumes and solvent mixes were evaluated for appropriate process performance. Finally, elution of retained metabolites was achieved with LC mobile phases during 5 min.

The composition of LC mobile phases was 5 mM of ammonium formate in water (phase A) and 5 mM of ammonium formate in methanol (phase B). Chromatographic gradient was programmed as follows with a constant flowrate of 0.5 mL min^−1^: from 85% of phase B, maintained during the initial 2 min, up to 100% of phase B in the next 5 min, conditions that were kept constant for the final 7 min of the chromatography. Furthermore, a post-run of 10 additional min was set to re-establish and equilibrate the initial conditions for the consequent run.

Chromatograph–detector interface parameters were set to 350°C and 9 L min^–1^ of drying gas (N_2_), a nebulizer pressure of 50 psi, and 4,500 V of capillary voltage. Detection was carried out in MRM mode. All metabolites detection parameters were studied by direct infusion of individual standard solutions at a concentration of 1 μg mL^−1^.

Assessment of analytical features of the proposed method was performed according to the Center for Drug Evaluation and Research (CDER) guidelines. Thus, linearity, sensitivity, accuracy, precision, and recovery were characterized. Applicability of the proposed method was also carried out for further use in clinical studies.

### Blood sampling and processing to isolate PBMCs

Venous blood from all patients was collected in tubes containing EDTA at baseline and 6 months after BS. PBCMs were isolated as previously described (43, 50).

### RNA extraction, quantification, and reverse transcription

Total RNA from PBMCs was isolated using Direct-zol RNA kit (Zymo Research, Irvine, CA, USA) following manufacturer’s instructions and as previously described (43, 50, 51). The amount of RNA recovered was determined and its quality assessed by the NanoDrop2000 spectrophotometer (Thermo Fisher). Specifically, all the RNA samples passed the quality controls, being 260/280 and 230/260 absorbance ratios among 1.8–2.0. As previously described (43, 52, 53), 1 μg of RNA was reverse transcribed (RT) to cDNA using random hexamer primers with the First Strand Synthesis Kit (Thermo Fisher).

### Analysis of components of the inflammasome machinery by qPCR dynamic array based on microfluidic technology

A 48.48 dynamic array based on microfluidic technology (Fluidigm, San Francisco, CA, USA) was developed and implemented to determine, simultaneously, the expression of 48 transcripts in 48 samples, following the same methods previously described (43, 54). Specific primers for human transcripts of the inflammasome machinery including NLR-/NOD-like receptors (n=7), regulators of inflammasome activation (n=15), cytokines and inflammation/apoptosis-related components (n=18), and cell-cycle and DNA-damage regulators (n=5) were used (42). In addition, three housekeeping genes were used. The selection of this panel of genes was based on two main criteria: 1) the relevance of the given inflammasome components and other cell cycle regulators in the inflammatory and apoptotic process and 2) the demonstrated implication in the inflammatory response in metabolic disorders, especially in OB conditions.

Preamplification, exonuclease treatment, and qPCR dynamic array based on microfluidic technology were implemented following manufacturer’s instructions using the Biomark System and the Real-Time PCR Analysis Software (Fluidigm), as previously described (55, 56). The expression level of each transcript was adjusted by a normalization factor (NF) obtained from the expression levels of two different housekeeping genes [beta actin (ACTB) and glyceraldehyde-3-phosphate dehydrogenase (GAPDH)] using Genorm 3.3. This selection was based on the stability of the housekeeping genes analyzed among the experimental groups to be compared, wherein the expression of these two housekeeping genes was not significantly different among groups.

### Statistical analysis

Between-group comparisons were analyzed by the Mann–Whitney U test (non-parametric data), or the Kruskal–Wallis test (non-parametric data, when we compared more than two groups). Paired analysis was performed by Student’s t (parametric data) or Wilcoxon test (non-parametric data). Chi-squared test was used to compare categorical data. Statistical analyses were performed using SPSS statistical software version 20 and Graph Pad Prism version 6. Heatmaps and clustering analysis were performed using MetaboAnalyst (57). Data are expressed as mean ± SD and percentages. p-values <0.05 were considered statistically significant.

## Results

Forty patients were evaluated, 55% were women, with a mean age of 45 years. As expected, patients underwent BS due to grade 3 OB (**Table 1**). Gastric bypass was the most common surgical procedure; only 17% received oral treatment with calcifediol before surgery. Six months after BS, patients presented a significant decrease in body weight and fat mass and increase in the percentage of lean mass (**Table 2**).

**Table 2:** Clinical characteristic of the evaluated patients (n=40) according to the presence of metabolic comorbidities at diagnosis.

Characteristic	Without metabolic comorbidities			With metabolic comorbidities		
	Baseline	6 months after bariatric surgery	*p*	Baseline	6 months after bariatric surgery	*p*
Age (years)	37 ± 7.7			49.6 ± 9		
BMI (kg/m^2^)	46.7 ± 4	34.6 ± 4.6	0.001	45.8 ± 6.8	34.1 ± 5	<0.001
Body weight (kg)	126.9 ± 1 5.5	93.5 ± 14.2	0.001	128.6 ± 25.2	97 ± 17.8	<0.001
Fat mass (%)	49.2 ± 4.4	31.7 ± 11.3	0.001	48.5 ± 5.2	33.3 ± 8.6	<0.001
Fat mass (kg)	62.2 ± 6.3	30.3 ± 11.1	0.001	61.9 ± 15.3	31.9 ± 11.2	<0.001
Lean mass (%)	52.9 ± 4.1	57.7 ± 8.6	0.2	52.5 ± 5.3	62.8 ± 6.2	0.068
Lean mass (kg)	61.8 ± 15.3	60.5 ± 13.8	0.3	65.6 ± 14	60.4 ± 14.8	0.005
Water (%)	38.4 ± 3.5	49.1 ± 9	0.001	38.9 ± 4.6	47.7 ± 6.8	<0.001
Water (Kg)	49.7 ± 10	45.7 ± 9.9	0.009	51.3 ± 12.6	44.5 ± 10	<0.001
Abdominal perimeter (cm)	133 ± 11	107 ± 9	0.001	138 ± 12.3	113.7 ± 11.2	<0.001
Serum 25-OHD (ng/dl)*	19.9 ± 8.1	28.1 ± 8.9	0.016	15.4 ± 5.9	29.6 ± 11	<0.001
Serum RCP (g/dl)	8.6 ± 4.4	2 ± 2	0.002	10 ± 7.3	4.3 ± 5.8	<0.001
Weight loss (%)	26.3 ± 5.7			25.9 ± 5.1		

*Measured using a chemiluminescence method.

### Changes in vitamin D metabolites in patients undergoing bariatric surgery

**Figure 1:**
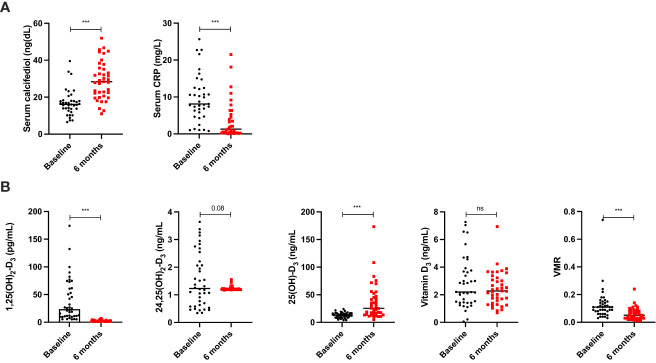
Change in serum levels of RCP and calcidiol using chemoluminiscence in patients before and 6 months after bariatric surgery **(A)**. Change in vitamin D metabolites using SPE-LC-MD/MS in patients before and 6 months after bariatric surgery **(B)**. Data represent the median ± interquartile range. Asterisks (***p<0.001) indicate significant changes between the clinical variables. ns, non-significant.

In clinical practice, routine evaluation of serum calcifediol and C-reactive protein (CRP) levels was performed during follow-up of patients with OB who undergo BS. As expected, serum levels of calcifediol (using a chemiluminescence method) increased 6 months after BS due to oral treatment, while CRP levels significantly decreased (**Figure 1A**). Calcium levels were normal in all patients (median, 8.7 mg/dl).

Using SPE-LC-MD/MS, 25(OH)-D_3_ levels also significantly increased, but most of the cases of extreme values (range, 4.96–173.2 ng/ml) were observed 6 months after BS when compared with the chemiluminescence method (range, 0.11–0.52 ng/ml). Remarkably, 24,25OH OH)_2_-D_3_ levels do not significantly change (**Figure 1B**). Additionally, 1,25(OH)_2_-D_3_ levels significantly decreased during follow-up. Despite 24,25(OH)_2_-D_3_ was not significantly decreased, VMR was markedly decreased 6 months after BS, in parallel with 1,25(OH)_2_-D_3_ levels (**Figure 1B**).

**Figure 2:**
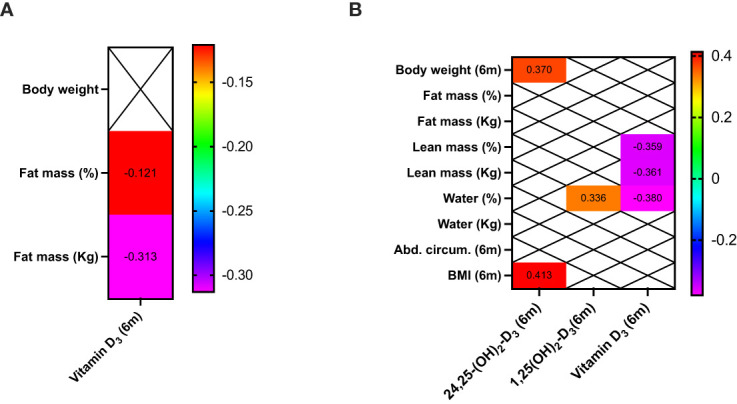
Clinical correlations between bioelectrical impedance, anthropometric measurements, and vitamin D metabolites in patients before **(A)** and 6 months after bariatric surgery **(B)**. Only significant correlations (p<0.05) are presented.

**Figure 2** shows that vitamin D_3_ levels (but not the other vitamin D metabolites) correlated with fat mass at baseline [median Body Mass Index (BMI), 46.7 kg/m^2^]. Six months after BS, vitamin D_3_ correlated with lean mass (median BMI, 36.6 kg/m^2^), after significant decrease in fat mass and increase in the percentage of lean mass (**Tables 1**, **2**).

### Vitamin D and metabolic comorbidities reversion after BS

**Figure 3:**
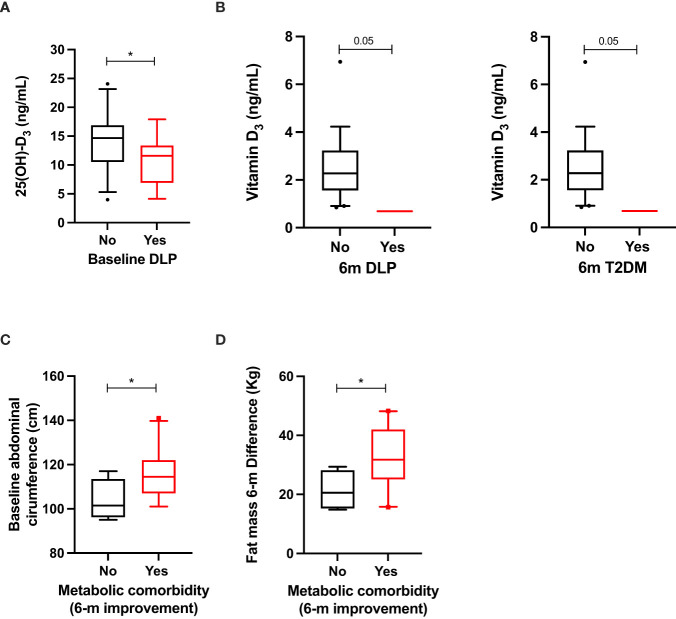
Significant clinical–molecular associations between vitamin D metabolites and the presence of baseline hypertension, dyslipidemia, and metabolic comorbidities in patients with OB before surgery **(A)**; clinical–molecular associations between vitamin D metabolites and improvement of hypertension and metabolic comorbidities 6 months after surgery **(B)**. Clinical associations between anthropometric measurements and improvement of metabolic comorbidities at baseline **(C)** and 6 months after surgery **(D)**. Data represent the median ± interquartile range. Asterisks (*p<0.05) indicate significant changes between the clinical variables.

Patients with baseline dyslipidemia (DLP) presented with lower 25(OH)-D_3_ levels compared with patients without such DLP (**Figure 3A**). Any other correlation between metabolic comorbidities and vitamin D metabolites was observed at baseline. Remarkably, patients in which DLP and T2DM did not reverse presented with lower vitamin D_3_ levels (**Figure 3B**).

When anthropometric variables were analyzed, improvement of metabolic comorbidities was associated with increased abdominal circumference at baseline and increased fat-mass difference (**Figures 3C, D**).

### Inflammasome is correlated with body composition and improvement of metabolic comorbidities after BS

**Figure 4:**
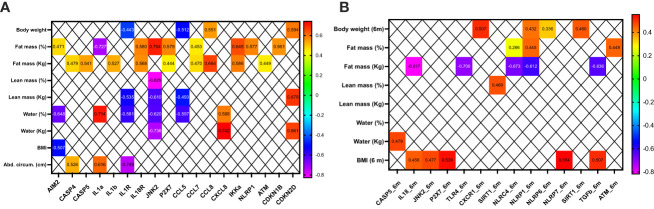
Clinical correlations between bioelectrical impedance, anthropometric measurements, and the mRNA expression of some inflammasome components in patients before **(A)** and 6 months after bariatric surgery **(B)**. Only significant correlations (p<0.05) are presented.

At baseline, fat mass was positively correlated with some key component of the inflammasome machinery, especially with inflammasome-activation components (AIM2, CASP5, CASP6, IL1b, IL18R, JNK2, and P2RX7), and also with the chemokines CCL7, CCL8, and with IKKA, and with the NOD-like receptor1 (NLRP1) (**Figure 4A**). A negative correlation was also observed with IL1a (**Figure 4A**). In contrast, lean mass was negatively correlated with IL1R, JNK2, and CCL5.

Six months after BS, fat mass was negatively correlated with IL18, TLR4, NLRC4, NLRP1, and TGFb. Fat mass did not correlate positively with any inflammasome component. In contrast, BMI positively correlated with IL18, JNK2, P2XR7, NLRP7, and TGFb (**Figure 4B**).

**Figure 5:**
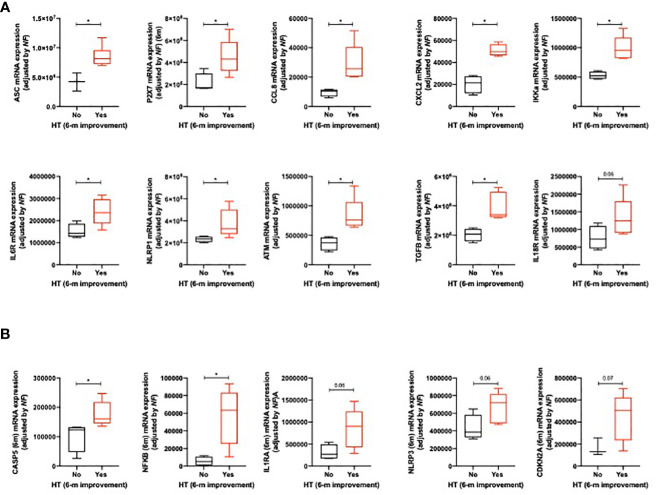
Significant clinical–molecular associations between the improvement of hypertension (6 months after surgery) and the mRNA expression of some inflammasome components at baseline **(A)** and 6 months after surgery **(B)**. Data represent the median ± interquartile range. Asterisks (*p<0.05) indicate significant changes between the clinical variables.

Patients who had improved hypertension 6 months after surgery presented with increased expression levels of the inflammasome activation components (ASC, P2X7, and IL18R), cytokines (CCL8, CXCL2, IKKA, and IL6R), NOD-like receptors (NLRP1), and cell cycle/DNA regulators (TGFb) (**Figure 5A**). These patients also presented with increased levels of CASP5 and NFKB and tended to express increased levels of IL1RA, NLRP3, and CDKN2A (**Figure 5B**).

**Figure 6:**
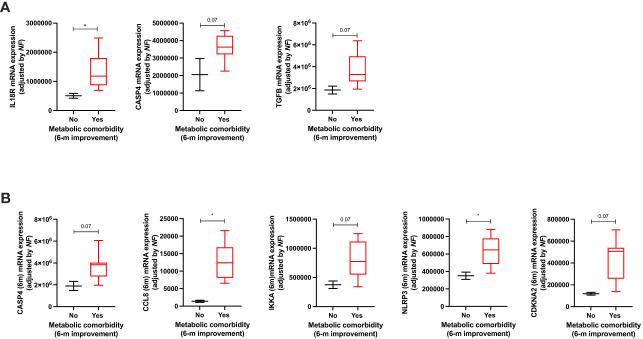
Significant clinical–molecular associations between the improvement of metabolic comorbidities and the mRNA expression of inflammasome components at baseline **(A)** and 6 months after surgery **(B)**. Data represent the median ± interquartile range. Asterisks (*p<0.05) indicate significant changes between the clinical variables.

When metabolic comorbidities were analyzed (hypertension, T2DM, and DLP), their improvement was associated with increased baseline levels of the inflammasome activation component IL18R (**Figure 6A**; CASP4 and TGFB tended to be also overexpressed in these patients). Additionally, the improvement of these metabolic comorbidities was also associated with increased expression levels of CCL8 and NLRP3 6 months after BS. Remarkably, CASP4, IKKA, and CDKN2A tended to be overexpressed in these patients (**Figure 6B**).

### Inflammasome and vitamin D metabolites are correlated in patients with obesity

**Figure 7:**
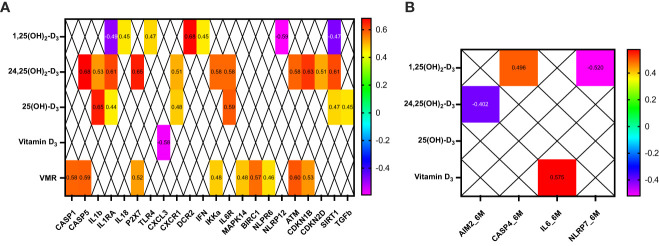
Clinical correlations between vitamin D metabolites and some inflammasome components in patients before **(A)** and 6 months after bariatric surgery **(B)**. Only significant correlations (p<0.05) are presented.

At baseline, 24,25(OH)_2_-D_3_ and VMR positively correlated with key inflammasome machinery components, especially with the inflammasome activation components (CASP1, CASP5, IL1b, IL1RA, and P2X7) and with cell cycle and DNA regulators (ATM, CDKN1B, CDKN2D, and SIRT1). In contrast, 25(OH)-D_3_ correlated with IL1b, IL1RA, CXCR1, IL6R, SIRT1, and TGFb (**Figure 7A**).

Six months after BS, 24,25(OH)_2_-D_3_ negatively correlated only with AIM2, and 25(OH)-D_3_ and VMR did not correlate with inflammasome after BS (**Figure 7B**).

## Discussion

In the present study, changes in VDES metabolites and relevant components of the inflammasome machinery (and inflammatory-associated factors) were comprehensively evaluated in a well-characterized cohort of patients with severe OB, who underwent BS and received treatment with oral calcifediol during 6 months. Additionally, their relation with metabolic comorbidities was studied. To the best of our knowledge, this is the first report that links important inflammasome components, VDES metabolites, and reversal of metabolic comorbidities in the clinical practice.

Decreased serum levels of 25(OH)-D_3_ have been widely reported in patients (of all ages) with OB (58, 59). In this cohort, decreased 25(OH)-D_3_ and vitamin D_3_ serum levels were associated with the presence of DLP at baseline and 6 months after BS, respectively. These results are in concordance with previous publications, in which vitamin D has been described as a significant independent inverse determinant of total cholesterol and low-density lipoprotein (LDL) and triglycerides (60). Despite this, no correlation or association was observed with lipoprotein (HDL) cholesterol levels, which have been previously described in the literature (61, 62). Remarkably, we did not observe differences in patients who underwent sleeve gastrectomy or gastric bypass, probably due to the fact that all patients received calcifediol at different dosage in order to achieve a sufficiency level (>30 ng/dl).

Additionally, some studies have suggested a link between medical treatment with vitamin D and changes in body composition. Specifically, a 3-month double-blind, randomized clinical trial in women demonstrated that vitamin D supplementation (25 μg/day as vitamin D3) reduced body fat mass regardless of BMI (63). In the same line, a meta-analysis described improvement in BMI and waist circumference following vitamin D supplementation (64). In contrast, we did not observe changes in BMI or body composition in patients according to the circulating 25(OH)-D_3_ levels at baseline. Similar results have been reported in intervention studies and meta-analyses in healthy population (65) and in patients with OB and after BS (66–68).

It is remarkable that despite a clear association between 25(OH)-D_3_ deficiency and OB, medical treatment has not been clearly associated with clinical improvement (69). This discordance might be explained by the metabolites measured. Indeed, it is well-known that 25(OH)-D_3_ is the most commonly measured metabolite (70), but it is frequently decreased in patients with OB due to several mechanisms. Several hypotheses have tried to explain this situation, including the 25(OH)-D_3_ sequestration by the adipose tissue, changes in vitamin D metabolism, volumetric dilution, or vitamin D receptor polymorphism (71). In this context, it would be valuable to determine another metabolite that could not be influenced by internal or external factors. A previous study of our group has demonstrated that the VMR was an appropriate marker of vitamin D status in patients with severe OB (grade 3 or higher) compared with patients without OB (23). In the present study, 6 months after surgery, serum levels of 25(OH)D significantly increased after oral supplementation with calcifediol, but 24,25(OH)_2_D_3_ levels did not change; these findings might be explained by the fact that patients received supplementation with calcifediol, which is a potent supplement with a higher rate of intestinal absorption (when compared with cholecalciferol) (72).

It has been previously described that the enlarged pool of visceral and subcutaneous adipose tissue probably impounds vitamin D and its metabolites, reducing their bioavailability (34). As expected, total levels of vitamin D_3_ were inversely correlated with fat mass at baseline in our cohort of patients, but this correlation disappeared 6 months after BS.

Curiously, vitamin D_3_ negatively correlated with lean mass after BS. Similar to this result, previous studies have suggested that there was no effect of vitamin D supplementation on lean mass and muscle strength in elderly subjects (73). Additionally, a double-blind, placebo-controlled randomized clinical trial that compared the administration of 2,000 IU/day oral vitamin D_3_ or placebo during a lifestyle-based weight loss intervention reported that vitamin D_3_ supplementation during weight loss decreased leg strength, without affecting lean mass, among postmenopausal women (74). Based on this, we suggest that an ideal study for evaluating the effect of vitamin D supplementation in patients with OB should include the measurement of VDES metabolites (or metabolome) and body composition evaluation in order to avoid confounding results.

As expected, our results demonstrated that inflammation status significantly decreased (reflected in decreased CRP levels) 6 months after BS and calcifediol treatment with normalization of 25(OH)D3 serum levels (in parallel to a significant decrease in body weight, fat mass, and reduction in metabolic comorbidities, as previously reported (75). In this context, improvement in metabolic comorbidities has been associated with fat mass loss after BS (66, 76, 77). In a previous study, we analyzed the effect of vitamin D supplementation on the reversal of metabolic comorbidities in a large cohort of patients after BS (n=346). In that cohort, improvement of comorbidities was independent of the serum levels of 25OHD (66). In contrast, in this study, patients that still presented with DLP or T2DM 6 months after surgery had lower circulating levels of vitamin D_3_ but no other metabolites. Differences might be explained by the different body composition of the evaluated patients (increased fat mass levels).

Previous studies have reported an association between inflammasome, OB, and fat mass (42, 78). In this cohort, we clearly observe positive correlations between fat mass and key inflammasome machinery components, and negative correlations between lean mass and inflammasome components, in concordance with previous publications that suggest a significant dysregulation of inflammasome components in patients with severe OB (37). For example, it has been described that visceral adipose tissue from patients with metabolic comorbidities show increased expression of IL1β (79), and IL18 has been associated with atherosclerosis and T2DM (80). These interleukins are clearly dysregulated and positively correlated with fat mass at baseline, but IL18 was negatively correlated with fat mass 6 months after BS and calcifediol treatment.

Additionally, the increased expression of inflammasome components in patients with severe OB has been associated with improvement of metabolic comorbidities, suggesting that there is a different inflammasome profile in patients that would significantly improve after BS. Particularly, according to previous studies, patients with essential HT exhibit elevated levels of circulating IL1β. In line with this, pyroptosis and inflammasome have been associated with the development of HT (42, 81). In our cohort, we observed that improvement of HT was associated with increased baseline levels of several key inflammasome components including IL6R, IL18R, and NLRP1.

Probably, NLRP3 is the most studied inflammasome. In fact, NLP3 has been associated with metabolic comorbidities including DLP and T2DM (82–84), and its role and regulation are a matter of debate for improving OB-related comorbidities (37). Remarkably, in our study, the patients with increased expression levels of NLRP3 improved HT and metabolic comorbidities 6 months after BS.

Finally, previous studies have demonstrated that NLRP3 can interact with vitamin D receptor (VDR) (35). Specifically, 1,25(OH)2 D and Vitamin D receptor signaling mechanisms increase the phagocytic ability of monocytes to modulate the innate immune system (85) and promote the ability of dendritic cells to modulate regulatory T-cell differentiation (86, 87). Furthermore, VDR deficiency is associated with increased inflammation and deregulation in several inflammatory diseases, such as inflammatory bowel disease, sepsis, diabetes, and asthma (87). It has been demonstrated that VDR inhibits the deubiquitination of NLRP3 by BRCC3 (35), which is a deubiquitinase with a crucial role for the post-transcriptional activation of NLRP3 (88). Similar results have been observed in peripheral blood monocytes from pregnant women with preeclampsia and in human monocyte cell lines (89). In addition, a preclinical model of OB and asthma showed a significant increase in airway hyper-responsiveness, airway inflammation, pro-inflammatory cytokine levels, mRNA expression of NLRP3 and IL1β in mice with OB, asthma, and lower 25(OH)D levels (90). In our cohort, IL1β and IL6R mRNA expression correlated with 25(OH)-D_3_ and 24,25(OH)_2_-D_3_ circulating levels in patients before BS.

*In vitro* studies have revealed that treatment with 1,25(OH)_2_D_3_ suppressed the expression of NLRP3 inflammasome-related genes and the production of IL1β in human corneal epithelial cells (91). 1,25(OH)_2_D_3_ treatment also inhibited caspase-1 activation and IL1β secretion in an ulcerative colitis model (92). Furthermore, treatment with 1,25(OH)_2_D_3_- and 25(OH)D_3_ also induced IL1β release from THP-1 cells *in vitro* (93). Additionally, it has been demonstrated that 1,25(OH)_2_VD_3_ inhibited the nigericin-induced activation step of NLRP1 inflammasome in unprimed keratinocytes (94).

Animal models have shown that treatment with 1,25(OH)2 D3 inactivates the NLRP3 inflammasome; in consequence, treatment with the hormonally active metabolite of VDES decreased surgery-induced neuroinflammation and memory and cognitive dysfunctions in aged mice (95). Treatment with 1,25(OH)2 D3 also exerted protective effects against retinal vascular damage and cell apoptosis especially in mice with type 1 diabetes (96). Furthermore, a strong correlation between increased NLRP3 inflammasome pathway and decreased 1,25(OH)2 D3 concentrations in the vitreous of proliferative diabetic retinopathy patients has been observed, suggesting that vitamin D supplementation may play a key role in preventing, treating, and improving the prognosis of this disease (97). In the present study, a significant reduction in the correlation between vitamin D metabolites and the molecular expression of inflammasome has been shown. Due to confounding factors, this change cannot be attributed to the medical treatment with calcifediol or the bariatric surgery-induced weight loss, exclusively.

This study has some limitations, including the cohort size, the short follow-up, and the absence of a control group for comparing the treatment with calcifediol; additionally, a specific evaluation of physical activity and lifestyle changes was not performed. Despite this, several strengths include the well-characterization of the cohort, the complete evaluation of the metabolites, and the clinical management of the patients according to the current clinical guidelines in BS.

Taken together, the results of the present study reveal new conceptual and functional information in the OB/BS field, with potential clinical implications, by demonstrating a clear dysregulation pattern of key components of the inflammasome machinery and VDES metabolism in patients with OB and in those patients who had improved metabolic OB-related comorbidities after BS and calcifediol treatment. This study provides solid evidence demonstrating that VDES metabolism in combination with some components of the inflammasome machinery might play a critical pathophysiological role in the improvement of OB-related comorbidities, offering a clinically relevant opportunity for novel targets that should be tested in humans. Importantly, these data demonstrated that the expression profile of inflammasome-machinery components can be dynamically modulated in PBMCs after BS and calcifediol treatment, suggesting that this profile could likely serve as a sensor and early predictor of the reversal of OB-related complications after BS. Interventional studies that evaluate vitamin D supplementation, vitamin D metabolites determination, and inflammasome expression should be performed in patients of different races and BMI.

## Data availability statement

The original contributions presented in the study are included in the article/supplementary material, further inquiries can be directed to the corresponding author/s.

## Ethics statement

The studies involving humans were approved by Reina Sofia University Hospital Ethics Committee. The studies were conducted in accordance with the local legislation and institutional requirements. The participants provided their written informed consent to participate in this study.

## Author contributions

ADH-M and RML. contributed to the design of the study; ADH-M contributed with the analysis and interpretation of data; FP-C, JMQG and RB performed a comprehensive analysis of the results and the manuscript; ADH-M. wrote the draft version of the article; LSC-P, MJM-P, AC-C, AM, JC, MCC, AC-D, MAG-M and MDG, contributed to the data acquisition and approved the final version of the article. All authors have read and agreed to the published version of the manuscript.
